# A 1-Minute Re-warm Up at High-Intensity Improves Sprint Performance During the Loughborough Intermittent Shuttle Test

**DOI:** 10.3389/fphys.2020.616158

**Published:** 2021-01-13

**Authors:** Takuma Yanaoka, Risa Iwata, Akane Yoshimura, Norikazu Hirose

**Affiliations:** ^1^Graduate School of Humanities and Social Sciences, Hiroshima University, Hiroshima, Japan; ^2^Graduate School of Sport Sciences, Waseda University, Saitama, Japan; ^3^Faculty of Sport Sciences, Waseda University, Saitama, Japan

**Keywords:** gastrointestinal temperature, muscle activation, intermittent team sports, half-time, heart rate

## Abstract

Although a 3- to 7-min re-warm up (RW) elicits performance and physiological benefits after half-time (HT), a time-efficient and feasible RW protocol is required for the use of an RW in the athletic setting. This study aimed to investigate the effect of a 1-min RW at high-intensity on the performance and physiological responses during the Loughborough Intermittent Shuttle Test (LIST). In a randomized and counterbalanced cross-over design, 12 male amateur intermittent team sports players (soccer, basketball, handball, and lacrosse; age, 22 ± 2 years; height, 1.70 ± 0.08 m; body mass, 65.1 ± 8.3 kg; body mass index, 22.4 ± 1.9 kg m^−2^; VO_2max_, 53.5 ± 4.5 ml kg^−1^ min^−1^) performed the LIST. The LIST comprised two 45-min halves separated by a 15-min HT. Each half comprised repetitions of exercise cycles consisting of 3 × 20-m walking, 1 × 20-m maximal sprint, 3 × 20-m jogging, and 3 × 20-m running. During the HT, the participants were assigned to a control trial (CON; 15-min seated rest) or an RW trial (1-min running at 90% of the maximal oxygen uptake after a 14-min seated rest). Compared to the CON, the RW prevents reductions in sprint performance at the fourth and sixth periods of the LIST (fourth: 2.4%, *p* = 0.002, *d* = 1.68, sixth: 3.6%, *p* = 0.012, *d* = 1.74) and a decrement of gastrointestinal temperature during HT (0.5°C, *p* = 0.010, *d* = 1.41). Moreover, the RW decreased the electromyogram amplitude of maximal voluntary contraction (MVC) after HT (12%, *p* = 0.017, *d* = 1.12) without a decrease of maximal voluntary contraction force, suggesting an increased neuromuscular efficiency (9%, *p* = 0.048, *d* = 0.58). The RW also increased the mean heart rate in the initial part of the second half (4 bpm, *p* = 0.016, *d* = 0.38). In conclusion, the RW improved sprint performance, core temperature, muscle activation, and heart rate in the second half of the LIST. The findings suggest that the RW should be recommended for intermittent team sports players when longer RWs are not possible.

## Introduction

The ability to perform a large amount of high-intensity running is one of the most important indicators for intermittent team-sport players. For example, the distance covered during high-intensity running has been related to outcomes in soccer matches (Chmura et al., 2018). However, intermittent team-sport players perform a lower amount of high-intensity running during the first 15 min of the second half compared to the first half (Mohr et al., 2005). This is especially remarkable because the players have half-time (HT) to assist them in recovering from the exertion of the first half. Lack of preparation for the second half is a suggested reason for the reduced amount of high-intensity running (Russell et al., 2015; Hammami et al., 2018; Silva et al., 2018), as the players warm-up before matches, but not during HT. Passive recovery during HT results in 1.1 and 2.0°C reductions in core and muscle temperatures, respectively (Mohr et al., 2004). These reductions in body temperature have been proposed as the primary mechanism for the reduced exercise performance after HT, because the elevated body temperature from the warm up is accompanied by increases in muscle metabolism, muscle fiber performance, and muscle fiber conduction velocity (McGowan et al., 2015). Besides exercise performance, it has been proposed that there is a high probability of muscle injuries (non-contact injuries) after HT (Rahnama et al., 2002) due to decreased muscle temperature during HT (Woods and Bishop, 2007) and from muscle strength deficiency before the commencement of the second half (Yamamoto, 1993; Rahnama et al., 2002). Previous studies have also reported that knee flexors are more fatigable (increased peak joint torque angle and electromyogram activity) than knee extensors and have been associated with hamstring muscle injuries (Coratella et al., 2015, 2018).

To protect against physiological changes and reductions in exercise performance due to passive recovery during HT, re-warm up (RW) strategies have been proposed (Russell et al., 2015; Hammami et al., 2018; Silva et al., 2018). Recent reviews have recommended RW at moderate-intensity for 5–7 min to protect against physiological changes and reductions in exercise performance during HT (Russell et al., 2015; Hammami et al., 2018; Silva et al., 2018). A previous study showed that a 7-min RW prevented 0.9 and 1.5°C reductions in core and muscle temperatures, respectively, and improved sprint performance by 3.9% compared to seated rest during HT (Mohr et al., 2004). Moreover, Lovell et al. (2013) reported that a 5-min RW prevented muscle strength deficiency compared to seated rest during HT. However, only 58% of players utilize this strategy because the RW duration of previous protocols exceeds a realistic time frame to be able to implement this strategy (Towlson et al., 2013). A previous study recommended within 3-min RW for practical application (Towlson et al., 2013), and recent studies reported the effectiveness of a 3-min RW on exercise performance (Yanaoka et al., 2018a,b; Fashioni et al., 2020). To make RW more applicable, it is necessary to develop an RW that players can perform in a short time-frame (within 1 min) of leaving the dressing room and the commencement of the second half.

Recent reviews have advocated the use of high-intensity RW if the protocol avoids inducing additional fatigue before the commencement of the second half (Hammami et al., 2018; Silva et al., 2018). Yanaoka et al. (2020) reported that cycling-based RW, irrespective of whether it comprised 1 min at high-intensity or 3 min at low-intensity, resulted in increased muscle temperature, oxygen uptake, and muscle activation, resulting in improved intermittent cycling sprint performance compared with seated rest. However, the lack of equipment (i.e., ergometers) in “away” stadiums is a major barrier to the administration of RW (Towlson et al., 2013). Thus, a time-efficient RW without equipment is needed. Moreover, the findings reported by Yanaoka et al. (2020) need to enhance ecological validity. The previous study employed 40 min of intermittent cycling exercise as a first half (Yanaoka et al., 2020), but there was a difference in physiological status after the first half between the previous study and that in actual intermittent team sports (Yanaoka et al., 2020). For instance, the core temperature in soccer players after the first half of matches is likely to be 39.0°C (Mohr et al., 2004), although the core temperature after 40 min of intermittent cycling exercise in the previous study was 38.0°C (Yanaoka et al., 2020). In addition, although the previous study assessed cycling sprint performance (Yanaoka et al., 2020), the correlation of sprint performance performed on a cycle ergometer with that performed on the ground is moderate at best (Fitzsimons et al., 1993). Sports-specific field tests need to be developed to enhance the ecological validity, separate to the contextual factors that exist in an actual match. The Loughborough Intermittent Shuttle Test (LIST) has been used by many studies (Saunders et al., 2012; Yanaoka et al., 2018c), and previous studies have suggested that the LIST simulates the activity pattern and the workload imposed by intermittent team sports (Nicholas et al., 2000; Magalhães et al., 2010; Coratella et al., 2016).

Another interesting aspect is whether the high-intensity RW could positively affect sprint performance in the last part of the second half. It has been demonstrated that intermittent team-sport players have a marked decline in the amount of high-intensity exercise in the last 15 min of the second half, because of decreased anaerobic capacity (Mohr et al., 2005). Edholm et al. (2015) proposed an association between RW and faster recruitment of the aerobic system because the RW used in the previous study increased heart rate (HR) after the commencement of the second half. HR is closely related to oxygen uptake responses during varying non-steady state exercise (Bot and Hollander, 2000). It has also been reported that high-intensity RW increased oxygen uptake at the start of the second half (Yanaoka et al., 2020). A high-intensity warm-up has been shown to improve exercise performance in the last part of the subsequent exercise, resulting from an increase in baseline oxygen uptake before subsequent exercise (Bishop, 2003a,b; McGowan et al., 2015). These findings suggest the possibility that high-intensity RW may decrease the initial oxygen deficit and leave greater anaerobic capacity (McGowan et al., 2015). However, it is yet to be determined whether high-intensity RW could prevent a decrement in sprint performance in the last part of the second half.

There is, therefore, a need to assess the effectiveness of time-efficient and practically applicable RW using sports-specific field tests, although 1-min cycling-based RW at high-intensity appears superior to seated rest for the prevention of physiological changes and reductions in exercise performance. The purpose of the present study was to investigate the performance (sprint and muscle strength) and physiological (core temperature, muscle activation, and HR) responses during the LIST after the implementation of an ecologically valid 1-min RW at high-intensity. We hypothesized that at least a 1-min RW at high-intensity would attenuate a reduction in core temperature and muscle activation during HT, maintaining sprint performance and hamstring strength after the commencement of the second half of a match.

## Materials and Methods

### Experimental Approach

**Figure 1:**
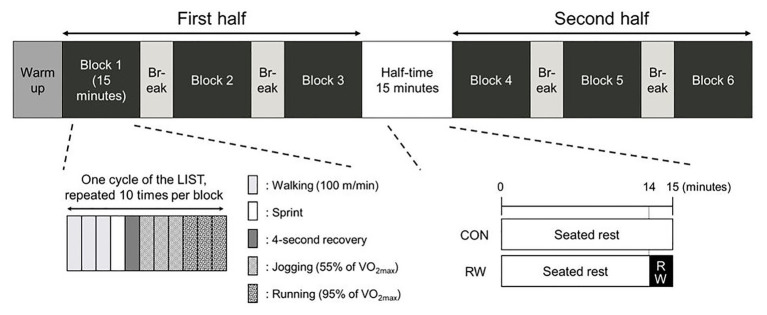
Schematic representation of the study protocol. CON: 15-min seated rest trial, RW: 1-min re-warm up at high-intensity trial, LIST: Loughborough Intermittent Shuttle Test.

To investigate the effect of 1-min RW at high-intensity on sprint performance, hamstring strength, core temperature, muscle activation, and HR, all participants completed two experimental sessions using a randomized and counterbalanced cross-over design after completing a preliminary visit to determine their maximal oxygen uptake (VO_2max_). All sessions were separated by at least 7 days and performed at the same time of day for each participant to avoid any circadian rhythm-related variations. In the experimental sessions, participants performed two 45-min halves of the LIST separated by a 15-min HT (Figure 1). The 15-min HT consisted of 15 min seated rest for the control (CON) trial and 1 min of RW at high-intensity after 14 min of seated rest for the RW trial. Experimental sessions were carried out in a thermoneutral environment (15.9 ± 2.4°C, 37.3 ± 9.3% relative humidity).

### Participants

A power calculation using cycling sprint performance data from a previous study (Yanaoka et al., 2020) was performed using a calculated effect size of 1.0, *α* = 0.05, and *β* = 0.2. This determined that 10 participants were required to demonstrate a difference in sprint performance. We chose to increase the number to 12. Therefore, 12 male amateur intermittent team-sport players participated in the present study (mean ± SD: age, 22 ± 2 years; height, 1.70 ± 0.08 m; body mass, 65.1 ± 8.3 kg; body mass index, 22.4 ± 1.9 kg m^−2^; and VO_2max_, 53.5 ± 4.5 ml kg^−1^ min^−1^). The participants were recruited from a university-based population, with a minimum of 5 years of intermittent team sports experience (soccer, basketball, handball, and lacrosse). The study was conducted after the end of the sports season, but all participants typically performed three to four training sessions each week. All participants recorded all meals and drinks consumed in the 24 h before each experimental trial and replicated their dietary intake in subsequent trials, ensuring standardization across all trials. Participants refrained from the intake of alcohol and caffeine for 24 h before each experimental trial, and fasted for 3 h, except for the consumption of water, before each experimental trial. The present study was approved by the Ethics Review Committee on Research with Human Subjects of Waseda University (approval number: 2017-287[1]), and all participants were informed of the benefits and risks of the investigation prior to signing an institutionally approved informed consent form.

### Procedures

Before the two experimental sessions, participants completed an incremental running test to determine VO_2max_. The exercise test used in the present study mimicked that of a previous study (Deighton et al., 2012), and comprised a 12-min submaximal incremental running test followed by an incremental uphill running test. The participants ran at 140 m min^−1^ on a 0% gradient, with 40 m min^−1^ increases in treadmill speed every 3 min until the speed reached 260 m min^−1^. After that, followed by a 10-min seated rest, they ran at an optional speed on 0% gradient, with 3% increases in treadmill gradient occurring every 2 min until volitional exhaustion. The optional speed was set at the speed corresponding to an HR of 150 beats min^−1^ or a rating of perceived exertion (Borg, 1982) of 12 on the submaximal exercise test. An automatic gas analysis system (AE 310 s, Minato Medical Science, Osaka, Japan) was used to measure oxygen uptake. The linear regression for oxygen uptake against running speed on 0% gradient was calculated and used to predict the relative exercise intensity during the LIST (55 and 95% of VO_2max_).

A schematic representation of the experimental design is shown in Figure 1. Before the commencement of the LIST, a standardized warm-up was conducted (involving 5 min of dynamic stretching, followed by 5 min of jogging, and then two repetitions of the exercise cycles of the LIST). After the warm-up, all participants were individually required to complete two 45-min halves of the LIST, separated by a 15-min HT. The LIST was designed to mimic the activity pattern and the workload of intermittent team sports (Nicholas et al., 2000; Magalhães et al., 2010; Coratella et al., 2016). The movement pattern of the LIST comprised 3 × 20 m at walking speed (100 m min^−1^), 1 × 20 m at a maximal running sprint, 4-s recovery, 3 × 20 m at a jogging speed corresponding to 55% of VO_2max_, and 3 × 20 m at running speed corresponding to 95% of VO_2max_. This exercise cycle was repeated 10 times during each exercise block, and the exercise block was repeated 3 times during each half. The exercise blocks were separated by 3-min breaks. These speeds during every 20 m iteration of the LIST was dictated by an audio signal.

For the RW trial, the initial 14 min of the HT were passive rest followed by a 1-min RW. The RW protocol comprised running between two lines, 20 m apart, at the speed corresponding to 90% of VO_2max_ (261 ± 19 m min^−1^). The speed during the RW was dictated by an audio signal. The RW intensity used in the present study was based on the previous study (Yanaoka et al., 2020). For the CON trial, participants were required to undertake passive rest by sitting on a chair for 15 min.

### Measurements

Nineteen-meter sprint times during the LIST were measured using telemetric photoelectric cells placed at a distance of 1 and 20 m (TCI system, Brower Timing System, Draper, United States). All participants were required to run as fast as they could to complete the 20-m distance. Mean sprint time was calculated in each exercise block during the LIST.

Two isometric maximal voluntary contraction (MVC) forces in the knee flexors of the right leg were measured by using a hand-held dynamometer (Micro FET 2, NIHON MEDIX, Chiba, Japan). The participants were in the prone position with hip and knee angles of 0° (full extension) for the MVCs (Kumazaki et al., 2012). The joint angles were selected because the knee flexion MVC torque with a knee angle of 0° was higher than that with a knee angle of 30°, 60°, and 90° in a previous study (Kumazaki et al., 2012). The upper limbs were crossed under the head. During the measurement, the hip and left leg were tightly secured by an operator. The duration of each contraction was 3 s. Participants were instructed to contract as quickly and powerfully as possible for each MVC. Verbal encouragement was given to all participants during the MVC. The two MVCs were separated by 30 s of passive rest. The dynamometer was placed on the Achilles tendon. The MVC force was determined as the mean of the highest force generated during each MVC. Before the first MVC test in each experimental trial, participants were required to complete 50 and 75% of the MVC in order to familiarize themselves with the test.

Gastrointestinal temperature as an indicator of core temperature was measured using an ingestible telemetric pill (VitalSense® Core Temperature Capsule, Equivital, New York, United States). The sensor transmitted a radio signal to an external receiver device (EQ02 LifeMonitor, Equivital, New York, United States) every 15 s. Participants were required to swallow the sensor 8 h before the experimental trials commenced. Ingestion of a pill sensor several hours before data collection (more than 8 h) has been shown to ensure a stable gastrointestinal temperature (Byrne and Lim, 2007). Owing to missing data, the gastrointestinal temperature data are presented for only five participants. The HR was monitored throughout the experimental trials using an HR monitor (Polar M430, Polar Electro, Kempele, Finland).

The electromyograms of the muscle bellies of the right biceps femoris were recorded during the MVC using a surface electrode (BioLog DL-5000, S&ME, Tokyo, Japan). The surface electrodes had a single differential configuration, an inter-electrode distance of 10 mm, and a 3-bar formation. To reduce impedance, the skin was abraded and washed before electrode placement. The electrodes were placed over the muscle belly of the biceps femoris, defined as half the distance between the ischial tuberosity and the lateral condyle of the tibia, and were taped to the skin using micropore tape (3 M Company, St Paul, MN, United States) to minimize movement artifact. The electrode placement was marked using a surgical marker to ensure the same repositioning of the electrode between the two trials. The EMG signals were amplified with a bandpass filter of 10–400 Hz at a sampling frequency of 1 kHz using the TRIAS system (TRIAS II, Q’sfix, Tokyo, Japan). An integrated electromyogram (iEMG) was calculated from a 1-s window between 3-s MVCs. The within-subject coefficient of variation for iEMG between two MVCs in the same measurement point was 7.4 ± 6.5%. Moreover, for each MVC, the neuromuscular efficiency as an indicator of peripheral muscle contractility was calculated (Mendez-Villanueva et al., 2012). The neuromuscular efficiency was calculated as follows: neuromuscular efficiency = MVC force/iEMG (Mendez-Villanueva et al., 2012). A higher ratio indicated a better neuromuscular efficiency in the present study.

Perceived fatigue was measured using the 11-point scale (Frzovic et al., 2000) before and after each half. The scale ranged from 0 to 10, with “0” defined as “absolutely no perceived fatigue” and “10” defined as “the worst perceived fatigue you have ever felt.”

### Statistical Analyses

Statistics were computed using SPSS software (version 25.0, SPSS Japan Inc., Japan). Statistical significance was set at *p* < 0.05. All values are shown as mean ± SD. The Shapiro-Wilk test was used to check for normality of distribution. All measurements were found to be normally distributed. Repeated measures two-factor analysis of variance was used to examine differences between the trials for all measurements. When significant interaction and main effect were found, the values were subsequently analyzed using a paired *t*-test or Bonferroni multiple comparisons test. Partial *η*^2^ values are also reported when significant interaction and main effect were found, with these classified as small (0.01–0.059), moderate (0.06–0.139), and large (≥0.14; Cohen, 1988). Moreover, the mean change during HT (*Δ*) was also calculated for sprint performance, MVC force, gastrointestinal temperature, iEMG, neuromuscular efficiency, and perceived fatigue. A paired *t*-test was used to examine the differences between the trials for *Δ* values. Analysis of all variables was also performed using Cohen’s *d* effect sizes with 95% CI, whereby >2.0 was categorized as a very large effect, 1.2–2.0 as a large effect, 0.6–1.2 as a moderate effect, 0.2–0.6 as a small effect, and 0.19 or lower as a trivial effect (Hopkins et al., 2009).

## Results

### Performance Index

**Figure 2:**
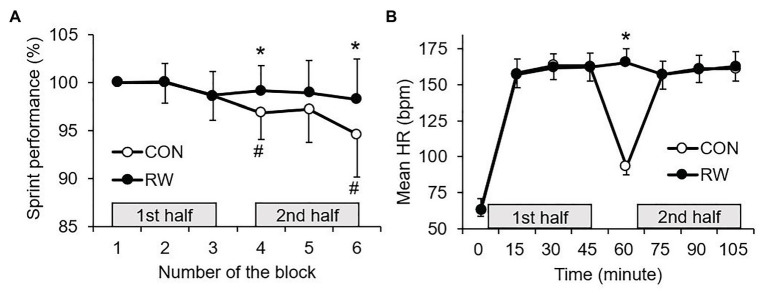
The mean sprint performance **(A)** and HR **(B)** between the two trials. CON: 15-min seated rest trial, RW: 1-min re-warm up at high-intensity trial (*n* = 12, mean ± SD). ^*^Significant difference between the trials (*p* < 0.05). ^#^Significant difference to the first block in the same trial (*p* < 0.05).

**Figure 3:**
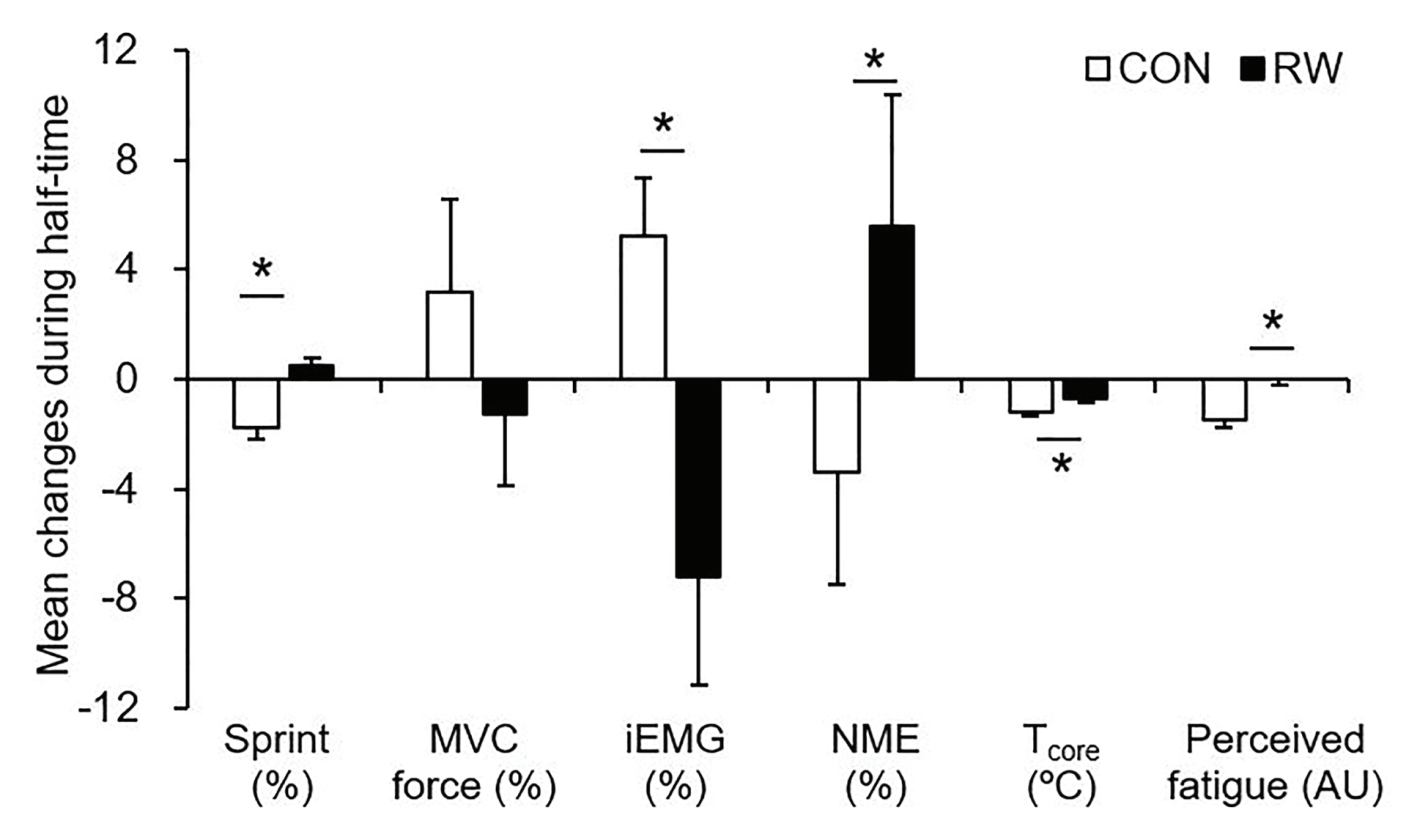
The mean changes during half-time of sprint performance, MVC force, iEMG, neuromuscular efficiency (NME), gastrointestinal temperature (T_core_), and perceived fatigue. CON: 15-min seated rest trial, RW: 1-min re-warm up at high-intensity trial [*n* = 12 (T_core_: *n* = 5), mean ± SD]. ^*^Significant difference between the trials (*p* < 0.05).

Figure 2A shows the mean changes of sprint performance between the trials, and Figure 3 shows the mean changes during HT of sprint performance, physiological measurements, and perceived fatigue between the trials. There was a trial × time interaction for the sprint performance (*p* = 0.009, partial *η*^2^ = 0.37). The mean sprint performances at the fourth and sixth exercise blocks were significantly reduced compared to that at the first exercise blocks in the CON trial (fourth: *p* = 0.028, Cohen’s *d* = 1.68, 95% CI: 0.3–6.1%; sixth: *p* = 0.021, Cohen’s *d* = 1.74, 95% CI: 0.6–10.2%), but not in the RW trial (*p* > 0.05). Furthermore, the mean sprint performances at the fourth and sixth exercise blocks were significantly higher in the RW trial than in the CON trial (fourth: *p* = 0.002, Cohen’s *d* = 0.89, 95% CI: 1.1–3.7%; sixth: *p* = 0.012, Cohen’s *d* = 0.84, 95% CI: 1.0–6.3%). *Δ* sprint performance was higher in the RW trial than in the CON trial (*p* = 0.001, Cohen’s *d* = 1.89, 95% CI: 1.3–3.4%, Figure 3). No significant difference between both trials was observed for the MVC and *Δ* MVC force.

### Physiological Index

Table 1 provides physiological measurements and perceived fatigue between the trials. There were no main effects of trial and trial × time interaction for the gastrointestinal temperature, iEMG, and neuromuscular efficiency. However, *Δ* gastrointestinal temperature and *Δ* neuromuscular efficiency were higher in the RW trial than in the CON trial (*Δ* gastrointestinal temperature: *p* = 0.010, Cohen’s *d* = 1.41, 95% CI: 0.2–0.8°C; neuromuscular efficiency: *p* = 0.048, Cohen’s *d* = 0.58, 95% CI: 0.1–17.1%, Figure 3). *Δ* iEMG was lower in the RW trial than in the CON trial (*p* = 0.017, Cohen’s *d* = 1.12, 95% CI: 2.7–22.1%, Figure 3).

**Table 1:** The gastrointestinal temperature, maximal voluntary contraction (MVC) force, integrated electromyogram (iEMG), neuromuscular efficiency, heart rate (HR), and perceived fatigue between the two trials.

Variables	Trial	Time (minute)							
		0	15	30	45	60	75	90	105
Gastrointestinal temperature (°C)	CON	37.3 ± 0.2	38.3 ± 0.5	38.8 ± 0.7	38.9 ± 0.5	37.7 ± 0.3	38.3 ± 0.4	38.7 ± 0.3	38.6 ± 0.4
	RW	37.3 ± 0.2	38.2 ± 0.6	38.6 ± 0.4	38.6 ± 0.4	38.0 ± 0.4	38.4 ± 0.6	38.7 ± 0.5	38.7 ± 0.4
MVC force (%)	CON	100 ± 0	-	-	91 ± 15	94 ± 14	-	-	86 ± 14
	RW	100 ± 0	-	-	92 ± 7	90 ± 11	-	-	93 ± 12
iEMG (%)	CON	100 ± 0	-	-	83 ± 8	88 ± 12	-	-	77 ± 15
	RW	100 ± 0	-	-	90 ± 14	83 ± 5	-	-	76 ± 11
Neuromuscular efficiency (%)	CON	100 ± 0	-	-	111 ± 21	107 ± 14	-	-	116 ± 31
	RW	100 ± 0	-	-	104 ± 16	110 ± 14	-	-	110 ± 16
Perceived fatigue (AU)	CON	2.3 ± 1.9	-	-	6.7 ± 1.0	5.2 ± 1.3	-	-	8.0 ± 1.2
	RW	1.9 ± 1.4	-	-	6.1 ± 0.8	6.1 ± 1.2	-	-	8.1 ± 1.1

CON: 15-min seated rest trial, RW: 1-min re-warm up at high-intensity trial [*n* = 12 (gastrointestinal temperature: *n* = 5), mean ± SD].

**Figure 4:**
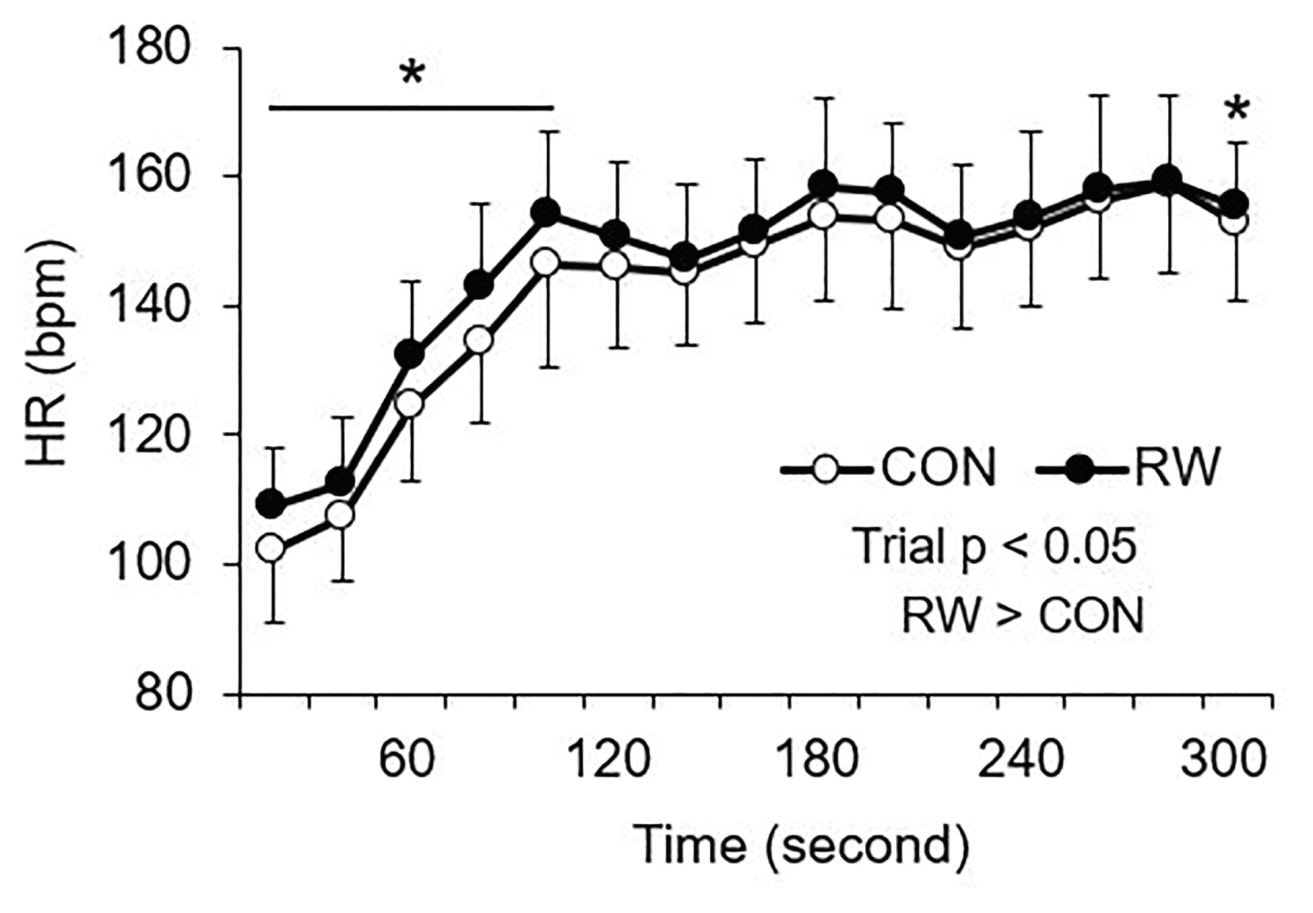
Changes in mean HR in the initial 5 min of the second half. CON: 15-min seated rest trial, RW: 1-min re-warm up at high-intensity trial (*n* = 12, mean ± SD). ^*^Significant difference between the trials (*p* < 0.05).

There was a trial × time interaction for HR (*p* < 0.001, partial *η*^2^ = 0.97, Figure 2B). The HR at the end of HT was significantly higher in the RW trial than in the control trial (*p* < 0.001, Cohen’s *d* = 8.87, 95% CI: 65.9–78.6 bpm). There was also the main effect of trial for mean HR during the initial 5 min of the second half (*p* = 0.013, partial *η*^2^ = 0.48, Figure 4). A subsequent *post hoc* test revealed that the mean HR in this part was higher in the RW trial than in the CON trial (*p* = 0.013, Cohen’s *d* = 0.38, 95% CI: 1.1–7.3 bpm).

### Perceived Fatigue

There were no main effects of trial and trial × time interaction for perceived fatigue (Table 1). The *Δ* perceived fatigue was lower in the RW trial than in the CON trial (*p* = 0.003, Cohen’s *d* = 2.11, 95% CI: 0.6–2.4 AU, Figure 3).

## Discussion

One-minute cycling-based RW appeared to elicit beneficial changes in intermittent cycling sprint and muscle activation (Yanaoka et al., 2020). However, there is a need to develop a practically applicable RW within 1 min and to investigate the effectiveness of this RW on performance and physiological responses using sports-specific field tests. In response, this study investigated whether a 1-min RW at a speed corresponding to 90% of VO_2max_ could prevent reductions in sprint performance and muscle strength and improve core temperature, muscle activation, and HR during the LIST. This study demonstrated the hypothesis, indicating that RW can prevent reductions in sprint performance compared to the CON trial. It is noteworthy that improvements in sprint performance in the initial and final 15-min during the second half were observed following RW, with moderate effect sizes identified. Moreover, the RW prevented declines in gastrointestinal temperature and neuromuscular efficiency during HT. The RW also increased HR after HT. However, there were no significant differences in HR and perceived fatigue at the end of the second half.

Sprint performance has a large impact on match performance. The ability to perform high-intensity exercise can affect match outcomes (Chmura et al., 2018), and the most frequent action during goal situations in professional soccer matches is straight sprinting (Faude et al., 2012). The improvements in sprint performance could, therefore, have potential performance benefits for intermittent team-sport players. In support of previous observations regarding RW, an improvement in sprint performance after RW was observed, and the magnitude of improvement in sprint performance is comparable to that noted in previous studies (moderate effect sizes; Edholm et al., 2015; Fashioni et al., 2020). Moreover, although previous studies have reported no beneficial influence of RW in sprint performance during the final part of the second half (Mohr et al., 2004; Lovell et al., 2013), the present study is, to the best of our knowledge, the first to observe that RW improves sprint performance in this period. The value of the present study is in showing that practically applicable RW (i.e., within a minute, without equipment, and need for little space) improves sprint performance. In matches, the majority of fitness coaches have acknowledged the physiological and performance benefits of RW, but only 58% of them administered RW (Towlson et al., 2013). This is due to a lack of time during HT and the lack of facilities and space available in the stadium (Towlson et al., 2013). Therefore, the RW used in this study has advantages in terms of both performance and applicability compared to the RW protocols used in the previous study (i.e., duration: 3–7 min, required equipment: machines for whole body vibration or leg press; Lovell et al., 2013; Zois et al., 2013; Fashioni et al., 2020).

While better sprint performance and the benefits of RW were observed, the MVC force was not affected by the RW. This result is inconsistent with that of a previous study which suggested that concentric hamstring strength during isokinetic contraction increased after 5-min RW (Lovell et al., 2013). This may be attributed to a methodological difference for assessing muscle strength between the present (isometric contraction) and previous (isokinetic contraction) studies (Lovell et al., 2013). The previous study suggested that both types I and II muscle fiber performances are affected by elevations in muscle temperature following the warm-up, however, type II fiber is more likely to benefit from increased muscle temperature (McGowan et al., 2015). Although an increase in muscle temperature accelerates the rate of enzymatic processes, including the adenosine triphosphatase activity, the rate of adenosine triphosphate hydrolysis is greater than that required by action when the contraction is isometric (Rall and Woledge, 1990). This extra energy is released as heat (Rall and Woledge, 1990). Thus, the warm-up effect may be beneficial when the action is dynamic, such as in isokinetic contraction. Moreover, a previous study reported that warm-up decreased the EMG amplitude during the MVC, but did not affect the MVC force (Stewart et al., 2003). These results are consistent with our findings that the RW decreased the iEMG and prevented a decline in neuromuscular efficiency during HT.

In accordance with the temperature benefit observed in previous studies on RW (Russell et al., 2015; Hammami et al., 2018; Silva et al., 2018), the present study revealed that the RW prevented a decline in gastrointestinal temperature with large effect sizes. Elevating body temperature by performing a warm-up was linked to faster adenosine triphosphate turnover, primarily *via* augmentation in the rate of creatinine phosphate utilization, as well as increases in anaerobic glycolysis and muscle glycogenolysis (McGowan et al., 2015). In addition, increased muscle fiber performance, muscle fiber conduction velocity, and neural transmission rates were observed under conditions of elevated body temperature (McGowan et al., 2015). A previous study reported that the decrease in body temperature at HT was correlated with the reduction in sprint performance during HT (Mohr et al., 2004). Thus, maintaining body temperature following the RW may contribute to improvements in sprint performance, as observed in the RW trial. However, the magnitude of improvement in gastrointestinal temperature observed in the present study is lower than that in a previous study in which participants undertook a 7-min RW at moderate-intensity (very large effect sizes; Russell et al., 2018). Further maintenance of body temperature is needed to elicit greater performance benefits because every 1°C reduction in muscle temperature is related to a 3% reduction in exercise performance (Sargeant, 1987). Russell et al. (2018) recently reported that a combination of RW and wearing heat maintenance garments helped in maintaining the gastrointestinal temperature compared to isolated strategies. Thus, in scenarios where longer duration RW is not possible, a combined strategy may offer a better maintaining body temperature. We have, however, no direct data to support these speculations and further study is thus required.

Another potential mechanism contributing to an improvement in sprint performance following the RW might be enhanced muscle activation. Another previous study (Blazevich and Babault, 2019) reported that high-intensity warm-up led to an enhanced voluntary muscular performance in subsequent exercise, a phenomenon called post-activation potentiation (Sale, 2002). The potential mechanisms underlying the post-activation potentiation phenomenon are an increase in the calcium sensitivity of the actomyosin complex caused by phosphorylation of the myosin regulatory light chain and an increase in higher-order motor neuron recruitment (Sale, 2002; Blazevich and Babault, 2019). A previous study reported that cycling-based RW at 90% of VO_2max_ led to enhanced muscle activation during subsequent cycling sprints (Yanaoka et al., 2020). Although muscle activation in sprints during the LIST was not measured in the present study, post-activation potentiation may have contributed to the improvement of sprint performance.

In the present study, the metabolic aspects of RW were considered using an assessment of HR. Elevated oxygen uptake following warm-up may decrease the initial oxygen deficit after the commencement of a subsequent exercise and spare finite anaerobic stores, potentially maintaining the ability to perform high-intensity exercises during the last part of a subsequent exercise (McGowan et al., 2015). In particular, a high-intensity warm-up (above the lactate threshold) increases baseline oxygen uptake before a subsequent exercise (Bishop, 2003a,b; McGowan et al., 2015). Although oxygen uptake was not directly measured in the present study, increases in HR at the end of HT and after the commencement of the second half were observed in the present study. These increases suggest that the RW elevated oxygen uptake in the initial part of the second half because a close relationship exists between HR and oxygen uptake responses during varying non-steady state activities (Bot and Hollander, 2000). This speculation is consistent with findings reported by Yanaoka et al. (2020), who observed that a high-intensity RW increased oxygen uptake and oxygen availability in the muscle compared to a passive rest during HT. Therefore, an improvement in metabolic aspects following high-intensity RW may contribute to maintaining sprint performance in the sixth block of the LIST.

Given that interference in the psychological preparation of players is one of the situational limiting factors in implementing an RW (Towlson et al., 2013), a study examining the psychological aspects of an RW is important in developing practical applications. In the present study, perceived fatigue did not decrease after RW compared to after the first half. Perceived fatigue was decreased in four participants after the RW compared with after the first half, and five participants showed no effects of perceived fatigue owing to the RW. Thus, there was individual variability for the influence of the RW on perceived fatigue, which should be considered in the actual matches. It could also be argued that high-intensity RW might result in accumulated fatigue. However, although HR was elevated after RW and perceived fatigue did not decrease during HT, significant differences in any physiological and psychological responses were not observed during the final part of the second half and at the end of the second half. Accordingly, the high-intensity RW did not induce additional fatigue, which is consistent with the findings of a previous study (Mohr et al., 2004; Lovell et al., 2013; Edholm et al., 2015).

Although it falls outside of the aim of the present study and is not investigated here, it is interesting to consider whether RW could reduce muscle injury risk during the start of the second half. A previous study reported an increased risk of muscle injury during this period, which resulted from decreased muscle temperature during HT (Woods and Bishop, 2007) and muscle strength deficiency at the end of HT (Yamamoto, 1993; Rahnama et al., 2002). Although the prevention of muscle strength deficiency following the RW was not observed in the present study, the RW did prevent a reduction in core temperature. Thus, it may be speculated that the RW reduces muscle injury risk, although this requires further study.

A limitation of the present study is the absence of muscle temperature measurements. Although the magnitude of changes is likely to differ between methods of temperature assessment, the response pattern during soccer-specific exercise appears similar between gastrointestinal and muscle temperatures (Mohr et al., 2004). Thus, muscle temperature may be maintained during HT by performing an RW. Moreover, the type of participants we recruited in the present study (amateur intermittent team-sport players) does not allow us to make a comparison with professional athletes. However, previous studies reported that the RW has a positive effect on sprint performance for amateur soccer players as well as professional soccer players (Mohr et al., 2004; Lovell et al., 2013; Edholm et al., 2015). Thus, it would be interesting to determine whether the RW used in the present study would have similar effects for professional athletes.

In conclusion, this study has demonstrated that a 1-min RW at high-intensity improved sprint performance, core temperature, muscle activation, and HR in the initial part of the second half during the LIST. Additional performance benefits were also observed and there was an improvement in sprint performance in the final part of the second half during the LIST. Moreover, the RW did not induce additional physiological and psychological fatigue in the final part of the second half. These findings suggest that the RW used in the present study may elicit performance and physiological benefits in the second half of intermittent team sports activities.

### Practical Applications

This study revealed that intermittent team-sport players may be able to improve their sprint performance using 1-min RW at high-intensity. This finding would support the use of an RW in the athletic setting, because the short-duration RW may be easily applied in actual matches and accommodate other specific ergogenic strategies during HT. It is feasible that an RW that involves running between two lines 20 m apart, as used in the present study, could be performed on the pitch after leaving the dressing room at HT. When RW cannot be performed on the pitch due to league policy (pitch protection and media regulations; Towlson et al., 2013), it may be performed using indoor facilities (such as a warm-up room) in stadiums. Although a lack of facilities and space in “away” stadiums could be a major barrier to the administration of RW (Towlson et al., 2013). The RW used in the present study is likely to have a practical application advantage because fitness coaches can administer it using an area of only 20 m, and without equipment.

## Data Availability Statement

The raw data supporting the conclusions of this article will be made available by the authors, without undue reservation.

## Ethics Statement

The studies involving human participants were reviewed and approved by the Ethics Review Committee on Research with Human Subjects of Waseda University (approval number: 2017-287[1]). The patients/participants provided their written informed consent to participate in this study.

## Author Contributions

TY designed the study with assistance from NH. TY, RI, and AY collected the data. All authors analyzed and interpreted the data, drafted, and revised the manuscript and figures, and approved the final published version.

### Conflict of Interest

The authors declare that the research was conducted in the absence of any commercial or financial relationships that could be construed as a potential conflict of interest.

## References

[ref1] Bishop D. (2003a). Warm up I: potential mechanisms and the effects of passive warm up on exercise performance. Sports Med. 33, 439–454. 10.2165/00007256-200333060-00005, PMID: 12744717

[ref2] Bishop D. (2003b). Warm up II: performance changes following active warm up and how to structure the warm up. Sports Med. 33, 483–498. 10.2165/00007256-200333070-00002, PMID: 12762825

[ref3] Blazevich A. J. Babault N. (2019). Post-activation potentiation versus post-activation performance enhancement in humans: historical perspective, underlying mechanisms, and current issues. Front. Physiol. 10:1359. 10.3389/fphys.2019.01359, PMID: 31736781 PMC6838751

[ref4] Borg G. A. (1982). Psychophysical bases of perceived exertion. Med. Sci. Sports Exerc. 14, 377–381. PMID: 7154893

[ref5] Bot S. Hollander A. (2000). The relationship between heart rate and oxygen uptake during non-steady state exercise. Ergonomics 43, 1578–1592. 10.1080/001401300750004005, PMID: 11083138

[ref6] Byrne C. Lim C. L. (2007). The ingestible telemetric body core temperature sensor: a review of validity and exercise applications. Br. J. Sports Med. 41, 126–133. 10.1136/bjsm.2006.026344, PMID: 17178778 PMC2465229

[ref7] Chmura P. Konefał M. Chmura J. Kowalczuk E. Zajac T. Rokita A.. (2018). Match outcome and running performance in different intensity ranges among elite soccer players. Biol. Sport 35, 197–203. 10.5114/biolsport.2018.74196, PMID: 30455549 PMC6234309

[ref8] Cohen J. (1988). Statistical power analysis for the behavioral sciences. Hillsdale: Lawrence Erlbaum Associates.

[ref9] Coratella G. Beato M. Schena F. (2016). The specificity of the Loughborough intermittent shuttle test for recreational soccer players is independent of their intermittent running ability. Res. Sports Med. 24, 363–374. 10.1080/15438627.2016.1222279, PMID: 27547994

[ref10] Coratella G. Bellin G. Beato M. Schena F. (2015). Fatigue affects peak joint torque angle in hamstrings but not in quadriceps. J. Sports Sci. 33, 1276–1282. 10.1080/02640414.2014.986185, PMID: 25517892

[ref11] Coratella G. Grosprêtre S. Gimenez P. Mourot L. (2018). Greater fatigability in knee-flexors vs. knee-extensors after a standardized fatiguing protocol. Eur. J. Sport Sci. 18, 1110–1118. 10.1080/17461391.2018.1469674, PMID: 29738677

[ref12] Deighton K. Zahra J. C. Stensel D. J. (2012). Appetite, energy intake and resting metabolic responses to 60min treadmill running performed in a fasted versus a postprandial state. Appetite 58, 946–954. 10.1016/j.appet.2012.02.041, PMID: 22366285

[ref13] Edholm P. Krustrup P. Randers M. B. (2015). Half-time re-warm up increases performance capacity in male elite soccer players. Scand. J. Med. Sci. Sports 25, e40–e49. 10.1111/sms.12236, PMID: 25048430

[ref14] Fashioni E. Langley B. Page R. M. (2020). The effectiveness of a practical half-time re-warm-up strategy on performance and the physical response to soccer-specific activity. J. Sports Sci. 38, 140–149. 10.1080/02640414.2019.1686941, PMID: 31680636

[ref15] Faude O. Koch T. Meyer T. (2012). Straight sprinting is the most frequent action in goal situations in professional football. J. Sports Sci. 30, 625–631. 10.1080/02640414.2012.665940, PMID: 22394328

[ref16] Fitzsimons M. Dawson B. Ward D. Wilkinson A. (1993). Cycling and running tests of repeated sprint ability. Aust. J. Sci. Med. Sport 25, 82–87.

[ref17] Frzovic D. Morris M. E. Vowels L. (2000). Clinical tests of standing balance: performance of persons with multiple sclerosis. Arch. Phys. Med. Rehabil. 81, 215–221. 10.1053/apmr.2000.0810215, PMID: 10668778

[ref18] Hammami A. Zois J. Slimani M. Russel M. Bouhlel E. (2018). The efficacy, and characteristics, of warm-up and re-warm-up practices in soccer players: a systematic review. J. Sports Med. Phys. Fitness 58, 135–149. 10.23736/S0022-4707.16.06806-7, PMID: 27901341

[ref19] Hopkins W. G. Marshall S. W. Batterham A. M. Hanin J. (2009). Progressive statistics for studies in sports medicine and exercise science. Med. Sci. Sports Exerc. 41, 3–12. 10.1249/MSS.0b013e31818cb278, PMID: 19092709

[ref20] Kumazaki T. Ehara Y. Sakai T. (2012). Anatomy and physiology of hamstring injury. Int. J. Sports Med. 33, 950–954. 10.1055/s-0032-1311593, PMID: 22895873

[ref21] Lovell R. Midgley A. Barrett S. Carter D. Small K. (2013). Effects of different half-time strategies on second half soccer-specific speed, power and dynamic strength. Scand. J. Med. Sci. Sports 23, 105–113. 10.1111/j.1600-0838.2011.01353.x, PMID: 21812822

[ref22] Magalhães J. Rebelo A. Oliveira E. Silva J. R. Marques F. Ascensão A. (2010). Impact of Loughborough intermittent shuttle test versus soccer match on physiological, biochemical and neuromuscular parameters. Eur. J. Appl. Physiol. 108, 39–48. 10.1007/s00421-009-1161-z, PMID: 19756713

[ref23] McGowan C. J. Pyne D. B. Thompson K. G. Rattray B. (2015). Warm-up strategies for sport and exercise: mechanisms and applications. Sports Med. 45, 1523–1546. 10.1007/s40279-015-0376-x, PMID: 26400696

[ref24] Mendez-Villanueva A. Edge J. Suriano R. Hamer P. Bishop D. (2012). The recovery of repeated-sprint exercise is associated with PCr resynthesis, while muscle pH and EMG amplitude remain depressed. PLoS One 7:e51977. 10.1371/journal.pone.0051977, PMID: 23284836 PMC3524088

[ref25] Mohr M. Krustrup P. Bangsbo J. (2005). Fatigue in soccer: a brief review. J. Sports Sci. 23, 593–599. 10.1080/02640410400021286, PMID: 16195008

[ref26] Mohr M. Krustrup P. Nybo L. Nielsen J. J. Bangsbo J. (2004). Muscle temperature and sprint performance during soccer matches—beneficial effect of re-warm-up at half-time. Scand. J. Med. Sci. Sports 14, 156–162. 10.1111/j.1600-0838.2004.00349.x, PMID: 15144355

[ref27] Nicholas C. W. Nuttall F. E. Williams C. (2000). The Loughborough intermittent shuttle test: a field test that simulates the activity pattern of soccer. J. Sports Sci. 18, 97–104. 10.1080/026404100365162, PMID: 10718565

[ref28] Rahnama N. Reilly T. Lees A. (2002). Injury risk associated with playing actions during competitive soccer. Br. J. Sports Med. 36, 354–359. 10.1136/bjsm.36.5.354, PMID: 12351333 PMC1724551

[ref29] Rall J. A. Woledge R. C. (1990). Influence of temperature on mechanics and energetics of muscle contraction. Am. J. Physiol. Regul. Integr. Comp. Physiol. 259, R197–R203. 10.1152/ajpregu.1990.259.2.R197, PMID: 2201213

[ref30] Russell M. Tucker R. Cook C. J. Giroud T. Kilduff L. P. (2018). A comparison of different heat maintenance methods implemented during a simulated half-time period in professional Rugby union players. J. Sci. Med. Sport 21, 327–332. 10.1016/j.jsams.2017.06.005, PMID: 28641863

[ref31] Russell M. West D. J. Harper L. D. Cook C. J. Kilduff L. P. (2015). Half-time strategies to enhance second-half performance in team-sports players: a review and recommendations. Sports Med. 45, 353–364. 10.1007/s40279-014-0297-0, PMID: 25504550

[ref32] Sale D. G. (2002). Postactivation potentiation: role in human performance. Exerc. Sport Sci. Rev. 30, 138–143. 10.1097/00003677-200207000-00008, PMID: 12150573

[ref33] Sargeant A. J. (1987). Effect of muscle temperature on leg extension force and short-term power output in humans. Eur. J. Appl. Physiol. Occup. Physiol. 56, 693–698. 10.1007/BF00424812, PMID: 3678224

[ref34] Saunders B. Sale C. Harris R. C. Sunderland C. (2012). Effect of beta-alanine supplementation on repeated sprint performance during the Loughborough intermittent shuttle test. Amino Acids 43, 39–47. 10.1007/s00726-012-1268-0, PMID: 22434182

[ref35] Silva L. M. Neiva H. P. Marques M. C. Izquierdo M. Marinho D. A. (2018). Effects of warm-up, post-warm-up, and re-warm-up strategies on explosive efforts in team sports: a systematic review. Sports Med. 48, 2285–2299. 10.1007/s40279-018-0958-5, PMID: 29968230

[ref36] Stewart D. Macaluso A. De Vito G. (2003). The effect of an active warm-up on surface EMG and muscle performance in healthy humans. Eur. J. Appl. Physiol. 89, 509–513. 10.1007/s00421-003-0798-2, PMID: 14551779

[ref37] Towlson C. Midgley A. W. Lovell R. (2013). Warm-up strategies of professional soccer players: practitioners’ perspectives. J. Sports Sci. 31, 1393–1401. 10.1080/02640414.2013.792946, PMID: 23734830

[ref38] Woods A. Bishop P. (2007). Warm-up and stretching in the prevention of muscular injury. Sports Med. 37, 1089–1099. 10.2165/00007256-200737120-00006, PMID: 18027995

[ref39] Yamamoto T. (1993). Relationship between hamstring strains and leg muscle strength. A follow-up study of collegiate track and field athletes. J. Sports Med. Phys. Fitness 33, 194–199. PMID: 8412057

[ref40] Yanaoka T. Hamada Y. Fujihira K. Yamamoto R. Iwata R. Miyashita M.. (2020). High-intensity cycling re-warm up within a very short time-frame increases the subsequent intermittent sprint performance. Eur. J. Sport Sci. 20, 1307–1317. 10.1080/17461391.2020.1713901, PMID: 31914360

[ref41] Yanaoka T. Hamada Y. Kashiwabara K. Kurata K. Yamamoto R. Miyashita M.. (2018a). Very-short-duration, low-intensity half-time re-warm up increases subsequent intermittent sprint performance. J. Strength Cond. Res. 32, 3258–3266. 10.1519/JSC.0000000000002781, PMID: 30199447 PMC6221412

[ref42] Yanaoka T. Kashiwabara K. Masuda Y. Yamagami J. Kurata K. Takagi S.. (2018b). The effect of half-time re-warm up duration on intermittent sprint performance. J. Sports Sci. Med. 17, 269–278. PMID: 29769828 PMC5950744

[ref43] Yanaoka T. Yamagami J. Kidokoro T. Kashiwabara K. Miyashita M. (2018c). Halftime rewarm-up with intermittent exercise improves the subsequent exercise performance of soccer referees. J. Strength Cond. Res. 32, 211–216. 10.1519/JSC.0000000000002197, PMID: 29257795

[ref44] Zois J. Bishop D. Fairweather I. Ball K. Aughey R. J. (2013). High-intensity re-warm-ups enhance soccer performance. Int. J. Sports Med. 34, 800–805. 10.1055/s-0032-1331197, PMID: 23444096

